# Geographical and Temporal Variations in Female Breast Cancer Mortality in the Municipalities of Andalusia (Southern Spain)

**DOI:** 10.3390/ijerph13111162

**Published:** 2016-11-22

**Authors:** Ricardo Ocaña-Riola, Carmen Montaño-Remacha, José María Mayoral-Cortés

**Affiliations:** 1 Escuela Andaluza de Salud Pública, Cuesta del Observatorio 4, 18011 Granada, Spain; 2 Instituto de Investigación Biosanitaria de Granada, Doctor Azpitarte 4, 4ª Planta, Edificio Licinio de la Fuente, 18012 Granada, Spain; 3 Servicio de Epidemiología y Salud Laboral, Dirección General de Salud Pública y Ordenación Farmacéutica, Secretaría General de Salud Pública y Consumo, Consejería de Salud de la Junta de Andalucía, Avenida de la Innovación s/n, Edificio Arena 1, 41020 Sevilla, Spain; mariac.montano.sspa@juntadeandalucia.es (C.M.-R.); josem.mayoral@juntadeandalucia.es (J.M.M.-C.)

**Keywords:** breast cancer, mortality, Geographical Information System, Bayesian analysis, small areas, spatial statistics, trend analysis, Andalusia, Spain

## Abstract

The last published figures have shown geographical variations in mortality with respect to female breast cancer in European countries. However, national health policies need a dynamic image of the geographical variations within the country. The aim of this paper was to describe the spatial distribution of age-specific mortality rates from female breast cancer in the municipalities of Andalusia (southern Spain) and to analyze its evolution over time from 1981 to 2012. An ecological study was devised. Two spatio-temporal hierarchical Bayesian models were estimated. One of these was used to estimate the age-specific mortality rate for each municipality, together with its time trends, and the other was used to estimate the age-specific rate ratio compared with Spain as a whole. The results showed that 98% of the municipalities exhibited a decreasing or a flat mortality trend for all the age groups. In 2012, the geographical variability of the age-specific mortality rates was small, especially for population groups below 65. In addition, more than 96.6% of the municipalities showed an age-specific mortality rate similar to the corresponding rate for Spain, and there were no identified significant clusters. This information will contribute towards a reflection on the past, present and future of breast cancer outcomes in Andalusia.

## 1. Introduction

Breast cancer is the most common cancer in women worldwide, both in terms of incidence and mortality. The GLOBOCAN series of the International Agency for Research on Cancer estimates that 1,677,000 new cases are now diagnosed annually, accounting for around 25% of total cancer cases in women. Additionally, 522,000 deaths from female breast cancer are reported annually, which represent 15% of cancer deaths worldwide [1]. These figures show a slight increase in both incidence and mortality over the last five years [2].

Around one in four new cases and one in four deaths from female breast cancer worldwide is registered in Europe. In 2012, this continent accounted for 463,800 new breast cancer cases and 131,200 deaths, which represents respectively 29% of total female cancer cases and 17% of female cancer deaths in European countries [3]. In Spain (southern Europe), the breast is the leading cancer site in women in terms of new cases and deaths. As a result, it is a major female health problem.

One of the health outcomes most frequently used to monitor breast cancer is mortality rate. Over the last years, a growing interest in the analysis of the temporal trend as well as in the study of geographical variation of breast cancer mortality has been seen in Europe due to population ageing, the need to plan healthcare systems and the need to improve the population’s health status. In addition, this interest has been accompanied by the availability of comprehensive mortality information systems.

Death rates of female breast cancer have declined severely over the last decades in Europe in response to advances in cancer therapy. As in other European countries, the trend in Spain is towards decreasing female mortality from breast cancer, especially since the mid-1990s [4]. With respect to geographical variations of mortality from breast cancer among European countries, the last published figures show that the age-standardized mortality rate ranged from 15 per 100,000 (Estonia) to 36 per 100,000 (Macedonia) in 2012. In Spain, the age-standardized mortality rate was 17 per 100,000 in 2012 [3]. As a result, Spain is in the group of European countries with the lowest mortality rates from breast cancer. These geographical variations in female breast cancer mortality could be caused by differences in incidence rates or by differences in survival, which is usually more favourable in more developed countries [1,3].

As differences in mortality could be due to differences in incidence or survival and, in turn, these differences could be caused by socioeconomic inequalities, geographical variations of mortality from female breast cancer can be present not only among countries, but among regions within a country. Administratively, Spain is divided into 19 self-governing regions, of which 17 are autonomous communities and two are autonomous cities (Ceuta and Melilla). The Spanish statistics concerning health determinants and health outcomes, including mortality from breast cancer, tend to vary by region [5,6]. As such, since the central government has returned healthcare responsibility to the autonomous regions, proper health planning requires analysis of mortality patterns in small-areas within each autonomous region.

**Figure 1:**
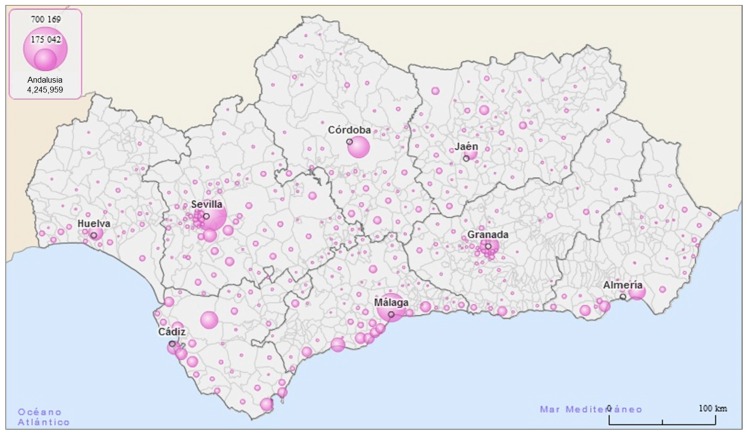
Andalusia (southern Spain) and its administrative division into eight provinces and 771 municipalities. Colored circles represent the female population of each municipality, which is directly proportional to the circle size.

Andalusia, one of the 17 autonomous communities in Spain, is located in the southern part of the Iberian Peninsula, bordered to the west by Portugal and to the south by the Atlantic Ocean and the Mediterranean Sea. With a surface area of 87,597 km^2^ and a population of 8,399,618 in 2015 (4,153,659 males and 4,245,959 females), it accounts for 17% of the Spanish territory and 18% of the Spanish population [5]. As the most populated and second largest autonomous region in Spain, it is comparable in extension and population to many European countries. Administratively, Andalusia is divided into eight provinces (Almeria, Cadiz, Cordoba, Granada, Huelva, Jaen, Malaga and Seville) and 771 municipalities, which are considered small geographical areas (Figure 1).

Spatial statistics applied to disease mapping is currently a major research area for studying the geographical variation of health outcomes in small areas. Advances in computer systems, the availability of powerful Geographical Information Systems (GIS) and the implementation of complex mathematical models in specialized software allow carrying out ecological small-area studies [7]. Here, hierarchical Bayesian models have played a leading role over the past few years, particularly the Besag–York–Mollié autoregressive conditional model [8].

Health outcomes in general, and mortality from female breast cancer in particular, are dynamic and most developed countries have experienced radical, rapid changes over the past few decades. Health determinants, healthcare technology and healthcare resources change over time and, in turn, also have repercussions for the population’s health outcomes. So, the evaluation of geographical variations of mortality from female breast cancer in small areas should be approached from a dynamic time perspective that is specific for each age group and calendar year, avoiding both grouping several annual data into a single period and using standardized mortality rates [9]. Perhaps, this is the best way to describe trends in geographical variations of mortality, to evaluate the repercussions of past health policies and to ascertain the current health status of the population so that future improvements may be undertaken.

Health policies for breast cancer based on evidence need research that provides a historical and dynamic image of the geographical variation of mortality in small areas from a spatio-temporal point of view. For this reason, the objectives of this paper are to describe the spatial distribution and the temporal evolution of the age-specific mortality rate from female breast cancer in the municipalities of Andalusia from 1981 to 2012, as well as comparison with the age-specific mortality rate in Spain.

## 2. Materials and Methods

### 2.1. Epidemiological Design and Scope

A small-area ecological study was devised using the municipality as the unit for analysis. The analysis was carried out on the 771 municipalities of Andalusia using annual information from 1981 to 2012, which forms a period of 31 years. The municipality boundaries have been constant over the entire time period.

### 2.2. Variables

The study considered three variables, whose information was recorded for each year and each of the following age groups: 15–44, 45–64, 65–74 and 75–84 years. These variables were the following:

- Mortality: Number of deaths from female breast cancer recorded in each municipality for each year and age group (International Classification of Diseases (ICD)-9 174 and ICD-10 C50).
- Population: Number of women living in each municipality for each year and age group.
- Spanish mortality rate: Number of deaths from female breast cancer per 10,000 women in Spain for each year and age group.

### 2.3. Sources of Information

Mortality data were derived from the Andalusian Institute of Statistics and Cartography, via the General Secretariat of Public Health and Consumer Affairs attached to the Ministry of Health of the Andalusian Regional Government.

The municipal population data for each year between 1981 and 2002 was taken from the population estimates provided by the National Statistics Institute (INE). From 2003 onwards, the residential population data was taken from the local census.

The specific mortality from female breast cancer in Spain for each age group and year, as well as the Spanish female population estimates between 1981 and 2002 were taken from INE data. From 2003 onwards, the data on the number of women in Spain was taken from the local census.

### 2.4. Statistical Data Analysis

Two spatio-temporal hierarchical Bayesian models were fitted for each age group. One of these was used to estimate the specific mortality rate in each municipality, together with its time trends from 1981 to 2012, and the other to estimate the specific rate ratio of each municipality compared with Spain as a whole (Table 1). Both models have different offsets. As a result, the second model is necessary to obtain appropriate rate ratio posterior distributions given that, in general, Bayesian smoothing of specific rate ratio is different to the ratio of smoothed specific rates [10].

In both models, it is assumed that the number of deaths observed within each age subgroup (*i*), municipality (*m*) and year (*t*) exhibit a Poisson distribution. The logarithm of the specific mortality rate and mortality rate ratio is expressed as the sum of a constant, a linear time effect, a quadratic time effect and two random spatial terms. One of these is unstructured and captures heterogeneity between municipalities, while the other is structured to account for the clustering of cases in space. Quadratic time effect captures most mortality trends [11], so it was included in Bayesian models. Possible cubic and upper time effects were tested using the Deviance Information Criterion (DIC) [12].

Both models include a spatio-temporal parameterization which enables the trend of the specific mortality rate and the rate ratio for each municipality to be modelled [13]. This estimate also pinpoints those geographical municipalities that have experienced an increase or decrease in mortality rates over time for each age group. Mathematical notation in Table 1 is usual in Conditional Autoregressive Models (CAR) with intrinsic CAR priors [8,13,14]. Gamma, flat and normal prior distributions were respectively used for precision parameters, intercept and model coefficients [13]. To avoid mathematical inconsistencies in mortality rate estimation, the value for the mortality variable was considered to be missing for all municipalities exhibiting both mortality and population equal to 0.

The model estimates were obtained by means of Markov Chain Monte Carlo (MCMC) algorithms, with 1000 iterations for burn-in and at least 10,000 later updates. The convergence was verified using two chains through the Gelman–Rubin method as modified by Brooks and Gelman [15]. WinBUGS software (MRC Biostatistics Unit, Cambridge, UK) was used for analysis [16].

**Figure 2:**
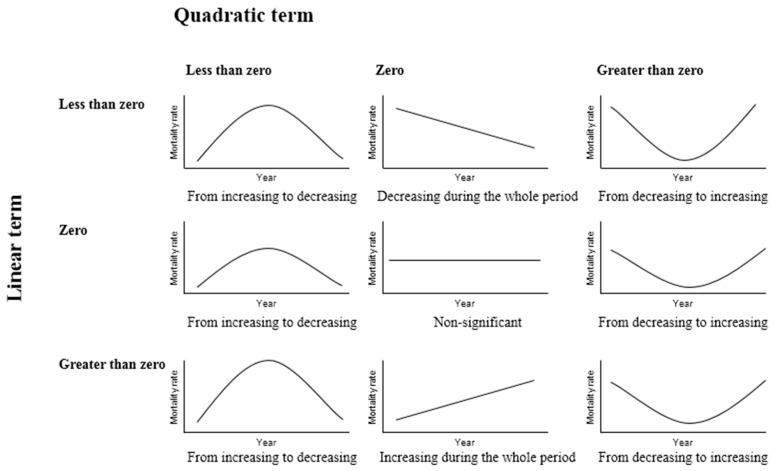
Possible graphical forms of the age-specific mortality trend for each municipality of Andalusia (southern Spain) according to the value of the linear and the quadratic term of its time function.

Concerning the temporal analysis of age-specific mortality, a significant trend was considered if the linear term or the quadratic term of the time function was greater or less than zero with a posteriori probability higher than 0.95. Thus, the trend was considered as decreasing during the whole period when only the linear term was less than zero with probability higher than 0.95. On the other hand, the trend was classified as increasing during the whole period when only the linear term was greater than zero with probability higher than 0.95. With regard to the quadratic form, the trend was considered as changing from increasing to decreasing when the quadratic term was less than zero with probability higher than 0.95, and it was considered to be changing from decreasing to increasing when the quadratic term was greater than zero with probability higher than 0.95. In other cases, the trend was considered non-significant (Figure 2).

To decide which municipalities had a mortality rate significantly higher than the Spanish mortality rate, we applied a decision rule based on computing the posterior probability that the specific rate ratio is greater than 1 with the following cut-off points: 0.05, 0.20, 0.80 and 0.95 [17]. Municipalities with values above 0.80 are small areas at risk. A higher significant mortality was considered when probability was greater than 0.95. Probabilities between 0.2 and 0.8 show little evidence that the rate ratio is above 1, so the specific mortality rate of these municipalities is considered to be similar to the reference mortality rate. Municipalities with values below 0.20 are low-risk small-areas and municipalities with probability lower than 0.05 are considered small-areas with a significantly lower specific mortality rate than the Spanish national rate.

Mortality mapping was conducted with Géoclip Server software (Emc3, Pechbonnieu, France).

## 3. Results

Between 1981 and 2012, 26,907 deaths from female breast cancer were recorded in all municipalities of Andalusia. Of these, 24,200 corresponded to the age groups analyzed in this study, with a frequency distribution as follows: 2677 (11.1%) deaths for the 15–44 year age group, 9998 (41.3%) deaths for the 45–64 year age group, 5900 (24.4%) deaths for the 65–74 year age group, and 5625 deaths (23.2%) for the group aged between 75 and 84 years old (Table 2).

**Figure 3:**
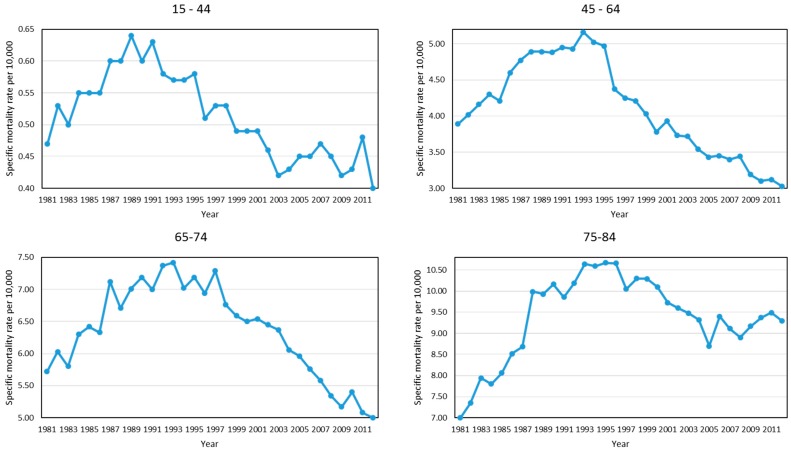
Trend of age-specific mortality rate per 10,000 women with respect to female breast cancer in Spain, 1981–2012.

In the following sections, the age-specific mortality from breast cancer in the municipalities of Andalusia will be contrasted with the age-specific death rates in Spain, which showed a quadratic form during the period 1981–2012 (Figure 3). Thus, Spanish age-specific mortality rates increased until the early 1990s and then declined until the end of the period. Only the group aged 75–84 years showed a slight increase of specific mortality rate from 2005 onwards.

### 3.1. Trend of the Age-Specific Mortality Rate in the Municipalities of Andalusia, 1981–2012

**Figure 4:**
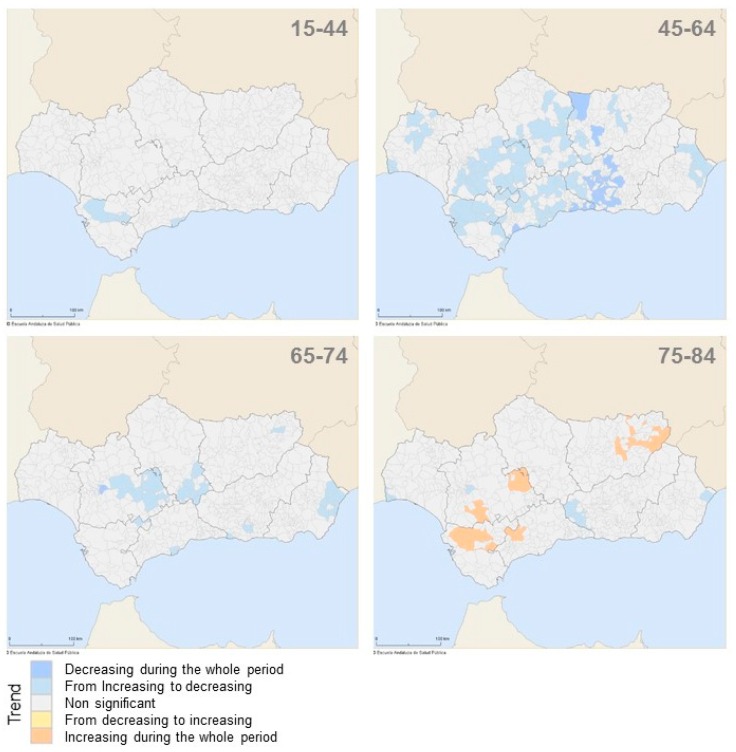
Trend of age-specific mortality rate from female breast cancer in the municipalities of Andalusia (southern Spain), 1981–2012.

During the period of study, all Andalusian municipalities exhibited a non-increasing mortality trend for all age groups under 74 years old because they showed one of these trend forms: diminishing during the whole period, from increasing to decreasing (like the quadratic form of the Spanish trend) or non-significant changes (mortality flat trend) (Figure 4). The same result was observed for the group aged 75–84 years, with the exception of 10 municipalities (1.3%) that showed a rising trend for that population group (Table 3A). These municipalities with increasing mortality trend from female breast cancer are scattered on the map of Andalusia and reproduce a similar trend to that shown by the Spanish mortality for the group aged 75–84 years during the last years of the period.

The group aged 45–64 years was shown in a higher number of municipalities (12.1%) with a quadratic form of the mortality rate trend (Table 3A), which increased until the mid-1990s and then declined until 2012.

### 3.2. Geographical Variation of the Age-Specific Mortality Rate in the Municipalities of Andalusia, 1981–2012

**Figure 5:**
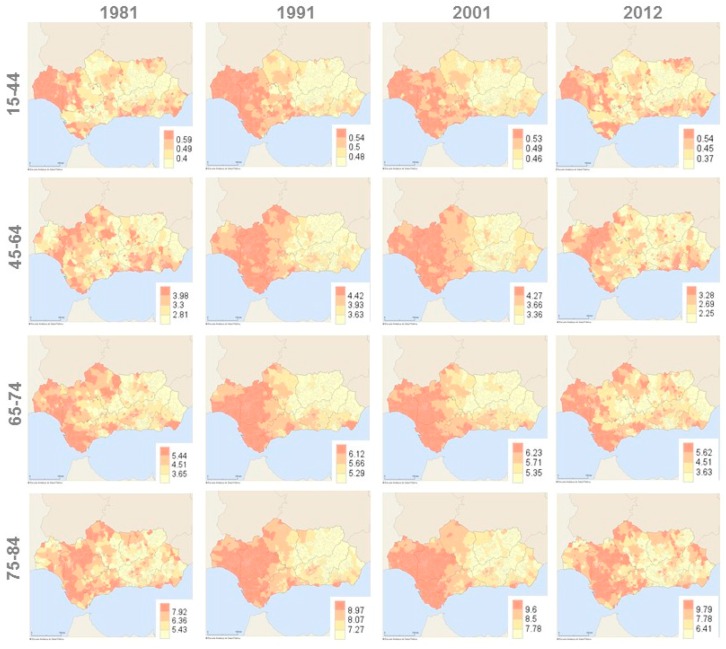
Geographical variations of age-specific mortality rate per 10,000 women from female breast cancer in the municipalities of Andalusia (southern Spain), 1981–2012.

Figure 5 shows the geographical distribution of the age-specific mortality rate from female breast cancer in Andalusia for the initial year of the study period (1981), the two middle years (1991, 2001) and the final year of the study (2012). This composition allows for monitoring of possible changes in geographical variations of mortality through sequential maps with decennial periodicity. Each map shows the division of the specific mortality rate into quartiles, using the darkest color for the last quartile values.

During the last decades of the 20th century, municipalities with higher age-specific mortality rates were grouped geographically in the western half of Andalusia. In 2012, some western municipalities were still positioned in the third quartile. However, geographical scattering of municipalities with higher mortality was now greater than it was in previous years (Figure 5). In addition, in spite of the fact that colors of the maps seem to show some geographical variations in breast cancer mortality, the analysis should be completed with the statistical summary of mortality rates (Table 3B). The similarity between quartiles of the estimated age-specific mortality rates suggests a certain degree of homogeneity in all Andalusian municipalities with respect to the breast cancer mortality in younger groups. Thus, in 2012, the interquartile range (difference between the third and first quartiles) stood at 0.17 deaths per 10,000 inhabitants in the group aged 15–44 years and at 1.03 per 10,000 inhabitants in the 45–64 year age group. This small variability suggests that geographical variations of breast cancer mortality are not epidemiologically relevant. For the groups aged 65–74 and 75–84 years, the interquartile range stood at 1.99 and 3.38 deaths per 10,000 inhabitants respectively. Although these are small variations, perhaps the difference between the municipality with the highest (maximum) and the lowest (minimum) estimated mortality rate suggests more geographical variability for these age groups. Similar results were found for the years before 2012.

### 3.3. Geographical Variation of the Age-Specific Mortality Rate Ratio in the Municipalities of Andalusia, 1981–2012

**Figure 6:**
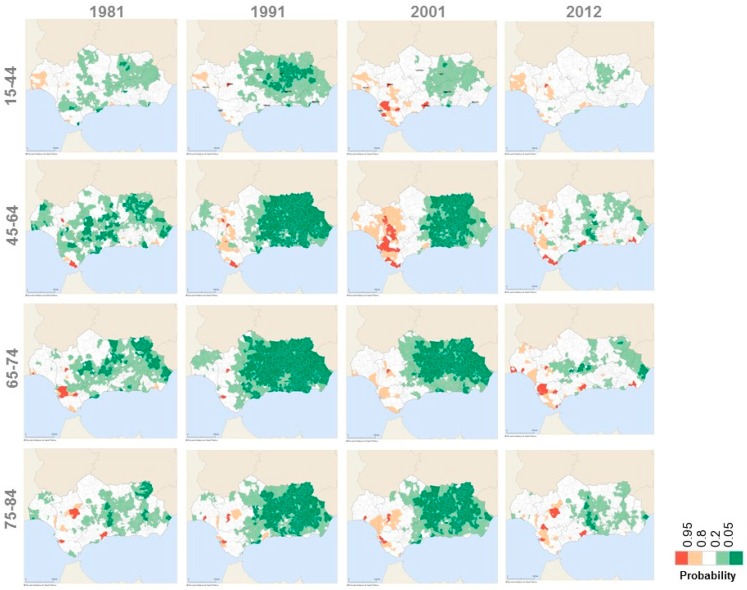
Geographical variations of the probability that the age-specific mortality rate ratio in each Andalusian municipality is greater than 1 using Spain as the reference, 1981–2012.

Figure 6 shows, with decennial periodicity, the geographical distribution of the probability that the age-specific mortality rate ratio in each Andalusian municipality is greater than 1, using Spain as the reference. The municipalities with probability greater than 0.95 are represented in dark red and the municipalities with probability less than 0.05 are shown in dark green. In other words, these colors respectively show those municipalities with an age-specific mortality rate significantly greater or significantly lower than the Spanish age-specific mortality rate. The other colors represent non-significant differences in mortality with respect to Spain.

During the last decade of the 20th century and the first years of the 21th century, there was a group of eastern Andalusian municipalities with age-specific mortality rates significantly lower than the age-specific national rates. These significant differences were gradually dissipated in the following years (Figure 6). In 2012, more than 96.6% of the Andalusian municipalities showed non-significant differences for all age groups respect to the Spanish mortality rates (Table 3C). The scarce municipalities that showed significant differences in the age-specific mortality rate with respect to Spain were scattered on the map of Andalusia, so no significant clusters were identified.

## 4. Discussion

In summary, the results of this study have shown that, between 1981 and 2012, 98% of the Andalusian municipalities exhibited or a decreasing or a flat mortality trend for all the age groups. In 2012, the variability of the estimated age-specific mortality rates was small among the municipalities of Andalusia, especially for population groups below 65. In addition, more than 96.6% of the municipalities showed an age-specific mortality rate similar to the corresponding for Spain, and there were not identified significant clusters.

The Spanish age-specific mortality rates increased until the early 1990s and then declined until 2012. A similar result was found from the mid-1990s in the 45–64 year age group for a number of Andalusian municipalities. This finding has also been observed in other European and Spanish studies on breast cancer mortality trend [3,18].

The ICD code changes that were implemented with ICD-10 in 1999, may have affected mortality trends. However, these changes did not affect breast cancer mortality in Spain, so the explanation for the reversal in mortality trend must be related to other factors [19]. Breast cancer screening might possibly account for this finding. In Spain, health policies to detect breast cancer began in 1990 [20]. In Andalusia, breast cancer screening began in 1995, with the aim of exploring women aged between 45 and 65 years. Currently, the coverage of the Andalusian screening program reaches around 78% of women between 50 and 69 years old. To ensure its effectiveness, participation should be above 70%. Right now, Andalusia is within the targets set in the recommendations of the European Cancer Council. The overall share is over 70% of women, with the exception of Almería (where a high number of immigrants who probably have not yet joined this program reside) and Seville (where private assistance serves many women that still decline public services). It is expected that women who have begun monitoring outside the public health system be integrated into the screening program in the near future [21]. As the screening program continues to have increasing coverage, a progressive mortality reduction in the next years for all the provinces and municipalities of Andalusia is expected. However, screening interventions have a time lag before a reduction in cancer mortality or other benefits are observed. Additionally, recent studies suggest that screening for breast cancer is most appropriate for patients with a life expectancy greater than 10 years [22]. Incorporating these considerations into screening guidelines would improve knowledge of the risks and benefits of screening for breast cancer. As a result, future research in Andalusia will show more accurately the effect of screening programs on breast cancer mortality.

In the present study, only 10 Andalusian municipalities (1.3%) showed a mortality rising trend of breast cancer mortality for the 75–84 year age group. These municipalities are scattered on the map of Andalusia and reproduce a similar trend to that shown by the Spanish mortality rates for this age group during the last years of the period. Similar results have been found in other Spanish studies [18]. One plausible explanation could be a different participation rate during the first years of breast cancer screening among the geographical areas from Andalusia. As a result, a smaller proportion of women may have been included in the breast screening program in the municipalities that showed increasing mortality trends. This hypothesis could be complemented by the results of previous studies related to the age-period-cohort effect on breast cancer mortality in Andalusia. The analysis of the cohort effect revealed a steady upward trend in breast cancer mortality risk for female generations born between 1896 and 1940, which could be more pronounced in some Andalusian municipalities for the population group that now is 75 or more years old [23].

On the other hand, the results of this study showed that during the last decade of the 20th century there was a group of eastern Andalusian municipalities with age-specific mortality rates significantly lower than the age-specific national rates. However, these significant differences gradually were dissipated in the following years. A plausible explanation could be the faster decrease in the age-specific mortality rates in Spain with respect to the eastern Andalusian municipalities. This phenomenon also has been observed in the ARIADNA surveillance system (Carlos III Health Institute), where the Spanish standardized mortality rate from breast cancer converges to the standardized mortality rate of the eastern provinces of Andalusia (Almeria, Cordoba, Jaén, Granada y Málaga) from the mid-1990s [24].

Usually, there is a long lag time between exposure to risk factors and development of breast cancer, so the rate of change of mortality could be due to a combination of multiple causes that cannot be completely differentiated in ecological studies. Family history, alterations in *BRCA1* or *BRCA2* genes, lifestyle, eating habits, consumption of alcohol, calorie intake, sedentary lifestyle, obesity and reproductive habits, such as the delay in childbearing age, hormonal contraceptive use, the reduction of the number of children and the abandonment of breastfeeding, are related to the risk of breast cancer [25]. Further, some studies suggest that primary care could play a fundamental role in awareness and early detection of breast cancer. In turn, improvements in the primary and hospital care system, including treatments, surgery radiotherapy, chemotherapy, hormonal therapy and adjuvant therapy to eliminate micrometastases, could reduce the mortality from incident cases [26]. Lastly, socioeconomic status has great impact in both incidence and mortality from breast cancer. The higher mortality in women with lower socioeconomic status seems to be explained by comorbidities, poorer lifestyles, lower participation in mammography screening and advanced stage at diagnosis [27]. With respect to this subject, Andalusia is the region with the highest unemployment in Spain and Europe, it is one of the regions of lower per capita income, and it shows, along with Extremadura, the lowest Human Development Index of the country [5,28], which could affect breast cancer mortality in comparison to the rest of Spain.

The prevalence of all these factors related to the incidence and mortality from breast cancer differ among autonomous communities, provinces and municipalities, so the interaction of all of these could explain the slower decrease in the age-specific mortality trend of the Andalusian municipalities with respect to Spain as a whole, as well as the small variation of age-specific mortality rates among the municipalities of Andalusia.

Due to the ecological design, all epidemiological studies in small-areas pose certain limitations that should be taken into account to be interpreted correctly [9].

Firstly, any statistical model allows a description of the geographical variation of mortality, but it cannot explain the differences in inter-municipal mortality nor the changes in intra-municipal mortality over time. As all data is aggregate, the level and time of exposure of any risk factor are unknown for deceased individuals. In addition, we cannot ascertain whether the number of deaths that have been registered in a given municipality correspond to individuals who have lived there most of their lives, and hence have been exposed to local environmental risk or prognosis factors. Therefore, any hypothesis suggesting a link between the higher or the lower mortality detected in any of the municipalities and social inequities, environmental exposure or use of healthcare services may lead to an ecological fallacy [29].

Secondly, in small-area epidemiology studies it is common to find an information bias linked to unregistered migrations that are not recorded by official information systems. Studies conducted in Spain show that some of deaths recorded in municipalities are for individuals who were not registered in the local census [30]. Similar facts have been shown in small-area studies conducted in Andalusia, and particularly for those aged 85 years or over, where an important number of municipalities presented a recorded number of deaths higher than the resident population [31]. Such flaws in information systems led us to reject the analysis of mortality for this population group in the present study. In addition, since 1975, deaths in Spain have been classified according to place of residence and not according to place of death. No study has been conducted to date in Andalusia to assess the quality of data regarding municipality of residence and cause of death recorded on the Death Statistics Form. As a result, we should be extremely careful when interpreting mortality maps, when undertaking studies to correlate geographical distribution with different indicators, and when positing hypotheses on the causes of death involved in differences in mortality between municipalities. Occasionally, inequalities in mortality recorded in small-area studies may only be the result of unregistered migratory flows that are not recorded in official population figures [30].

Finally, the denominator of the age-specific mortality rate must be the number of people who run the risk of death. In the study of mortality from female breast cancer, the denominator should not be the whole female population in the given geographic area but only those women who were alive during the study period following a diagnosis of breast cancer, i.e., the prevalent cases. As in most research, the present study uses the whole female population as the denominator instead of the number of prevalent cases because municipality data from the Cancer Registry of Andalusia are not yet available and there is no Spanish Cancer Registry. As a result, the real age-specific mortality rate in municipalities and Spain as a whole could be underestimated [9]. The lower the breast cancer prevalence in the geographic area, the greater is the underestimation. In addition, the calculation of age-specific rates ratio using the total female population as the denominator gives rise to skewed results regarding the real rates ratio. The bias factor is equal to the quotient between prevalence for disease in both areas, so the age-specific mortality rates ratio will be correct only if Spain and each Andalusian municipality have the same disease prevalence [9]. This was the assumption of the present study. However, if this hypothesis is not correct, the results could suggest unfounded geographic inequities in mortality.

Taking into account these comments, we have to be very cautious before suggesting any epidemiological cluster concerning mortality from female breast cancer. The higher or lower mortality observed in some municipalities could be the consequence of different lifestyles, socioeconomic status or contextual factors. However, it also may only be the result of unregistered migratory flows or the effect of using certain denominators in the estimation of mortality rates and mortality rates ratio. Given that death is the endpoint of a past health record, ecological studies from mortality indicators should be complemented by individual-based studies that would provide an in deep view of the population’s health status.

Dynamic mortality maps are able to provide up-to-date understanding of the geographical variation and evolution over time of mortality from breast cancer and other causes of death, substantially improving on the information provided by conventional static atlases [32]. However, a systematic validation of the information sources, a careful statistical analysis and a thorough interpretation of mortality maps are all still needed in epidemiological research. By building this careful practice into future research, we will generate a useful procedure for health policy planning.

The present paper is a continuation of our research line on spatio-temporal models for the study of temporal evolution and geographical distribution of health outcomes. The reader interested in the published studies on other causes of death in Andalusia, or methodological aspects related to disease mapping, can obtain additional information from the references at the end of this paper [9,23,30,31,32], as well as at the DEMAP web site [33]. The conclusions of these studies have been cited, in part, during the discussion of this paper and together can help to understand the evolution of mortality in Andalusia over both space and time.

## 5. Conclusions

The results of this study have shown that, between 1981 and 2012, 98% of the Andalusian municipalities exhibited or a decreasing or a flat mortality trend for all the age groups. In 2012, the variability of the estimated age-specific mortality rates was small among the municipalities of Andalusia, especially for population groups below 65 years of age. In addition, more than 96.6% of the municipalities showed an age-specific mortality rate similar to the corresponding rate for Spain, and there were no identified significant clusters. Since the screening program continues to increase its coverage, a progressive mortality reduction is expected in the next years for all the provinces and municipalities of Andalusia. However, screening interventions have a time lag before a reduction in cancer mortality or other benefits are observed. Future research in Andalusia will show more accurately the effects of both screening programs as well as other contextual factors on breast cancer mortality.

## Figures and Tables

**Table 1:** Modelling spatio-temporal distribution of mortality from female breast cancer in small-areas of Andalusia (southern Spain).

	Specific Mortality Rate	Specific Mortality Rate Ratio
**Distribution**	$o_{imt}$~$Poisson\left(\mu_{imt}\right)$	$o_{imt}$~$Poisson\left(\mu_{imt}\right)$
**Mean**	$\mu_{imt}=p_{imt}r_{imt}$	$\mu_{imt}=\left(p_{imt}R_{it}\right)RR_{imt}$
**Model**	$\log\left(r_{imt}\right)=\beta_{0i}+\left(\beta_{1i}+\delta_{1im}\right)\left(t-\bar{t}\right)+\left(\beta_{2i}+\delta_{2im}\right){\left(t-\bar{t}\right)}^{2}+u_{im}+v_{im}$	$\log\left(RR_{imt}\right)=\beta_{0i}+\left(\beta_{1i}+\delta_{1im}\right)\left(t-\bar{t}\right)+\left(\beta_{2i}+\delta_{2im}\right){\left(t-\bar{t}\right)}^{2}+u_{im}+v_{im}$
**Constant terms**	$\beta_{0i}$~*flat*, $\beta_{1i}$~N (0, 0.00001), $\beta_{2i}$~N (0, 0.00001)	
**Temporal random structure**		
*Structured effect*	$\left[\delta_{aim}|\delta_{ain},\;m\neq n\right]$~$N\;\left({\bar{\delta }}_{aim},\;1/\gamma_{aim}\right)$, ${\bar{\delta }}_{aim}=\frac{\sum_{n\neq m}\omega_{mn}\delta_{ain}}{\sum_{n\neq m}\omega_{mn}},\;\gamma_{aim}=\frac{\gamma_{\delta_{ai}}}{\sum_{s\neq t}\omega_{mn}}$, *a* = 1, 2	
*Hyperparameter*	$\gamma_{\delta_{ai}}$~Gamma (0.5, 0.0005), *a* = 1, 2	
**Spatial random structure**		
*Structured effect*	$\left[u_{im}|u_{in},\;m\neq n\right]$~$N\left({\bar{u}}_{im},\;1/\lambda_{im}\right)$, ${\bar{u}}_{im}=\frac{\sum_{n\neq m}\omega_{mn}u_{in}}{\sum_{n\neq m}\omega_{mn}},\;\lambda_{im}=\frac{\lambda_{u_{i}}}{\sum_{s\neq t}\omega_{mn}}$	
*Non-structured effect*	$v_{im}$~$N\left(0,\;1/\lambda_{v_{i}}\right)$	
*Hyperparameters*	$\lambda_{u_{i}}$~Gamma (0.5, 0.0005) $\lambda_{v_{i}}$~Gamma (0.5, 0.0005)	
**Notation**	$o_{imt}$: deaths within age subgroup *i* in municipality *m* at time *t*	$p_{imt}$: population within age subgroup *i* in municipality *m* at time *t*
	$r_{imt}$: specific mortality rate in municipality *m* at time *t*	$R_{it}$: specific mortality rate in Spain at time *t*
	$\bar{t}$: Median year	$RR_{imt}$: specific rate ratio of municipality *m* compared with Spain at time *t*
	$\omega_{mn},\;m\neq n$: adjacency matrix ($\omega_{mn}=1$ if *m* and *n* are neighbour areas and $\omega_{mn}=0$ otherwise)	

**Table 2:** Summary of mortality from female breast cancer in Andalusia (southern Spain) every ten years and for the full period (1981–2012), by age groups.

**Age Group**	**15–44**					**45–64**				
**Year**	**1981**	**1991**	**2001**	**2012**	**1981–2012**	**1981**	**1991**	**2001**	**2012**	**1981–2012**
Deaths from female breast cancer	71	106	102	79	2677	268	348	340	319	9998
Female population	1,301,212	1,552,496	1,727,353	1,771,291	52,076,904	673,832	716,606	761,630	1,050,092	25,077,188
Age-specific mortality rate per 10,000	0.55	0.68	0.59	0.45	0.51	3.98	4.86	4.46	3.04	3.99
**Age group**	**65–74**					**75–84**				
**Year**	**1981**	**1991**	**2001**	**2012**	**1981–2012**	**1981**	**1991**	**2001**	**2012**	**1981–2012**
Deaths from female breast cancer	159	190	262	197	5900	105	159	205	271	5625
Female population	238,227	272,957	342,834	347,981	9,672,430	124,954	162,496	200,940	281,236	6,129,090
Age-specific mortality rate per 10,000	6.67	6.96	7.64	5.66	6.10	8.40	9.78	10.20	9.64	9.18

**Table 3:** Age-specific mortality trend, age-specific mortality rate and age-specific mortality rates ratio from female breast cancer in the municipalities of Andalusia, 1981–2012.

**A**	**15–44**				**45–64**				**65–74**				**75–84**			
**Age-specific mortality trend**	**Municipalities**		**Percentage**		**Municipalities**		**Percentage**		**Municipalities**		**Percentage**		**Municipalities**		**Percentage**	
Decreasing during the whole period	0		0.0%		39		5.0%		1		0.1%		0		0.0%	
From increasing to decreasing	7		0.9%		93		12.1%		29		3.8%		6		0.8%	
Non-significant	764		99.1%		639		82.9%		741		96.1%		755		97.9%	
From decreasing to increasing	0		0.0%		0		0.0%		0		0.0%		0		0.0%	
Increasing during the whole period	0		0.0%		0		0.0%		0		0.0%		10		1.3%	
**B**	**15–44**				**45–64**				**65–74**				**75–84**			
**Age-specific mortality rate per 10^4^**	**1981**	**1991**	**2001**	**2012**	**1981**	**1991**	**2001**	**2012**	**1981**	**1991**	**2001**	**2012**	**1981**	**1991**	**2001**	**2012**
Maximum	2.71	0.72	0.68	2.58	8.35	5.72	5.63	8.54	13.76	8.46	7.92	13.73	18.43	12.67	13.76	26.20
Percentile 75	0.59	0.54	0.53	0.54	3.98	4.42	4.27	3.28	5.44	6.12	6.23	5.62	7.92	8.97	9.60	9.79
Percentile 50	0.49	0.50	0.49	0.45	3.30	3.93	3.66	2.69	4.51	5.66	5.71	4.51	6.36	8.07	8.50	7.78
Percentile 25	0.40	0.48	0.46	0.37	2.81	3.63	3.36	2.25	3.65	5.29	5.35	3.63	5.43	7.27	7.78	6.41
Minimum	0.19	0.42	0.40	0.19	1.54	2.96	2.91	1.23	1.96	4.08	4.44	1.70	3.16	5.87	6.78	3.43
Interquartile range	0.19	0.06	0.07	0.17	1.17	0.79	0.91	1.03	1.89	0.83	0.88	1.99	2.49	1.70	1.82	3.38
Spanish age-specific mortality rate	0.47	0.63	0.49	0.40	3.89	4.95	3.93	3.03	5.72	7.00	6.54	5.00	7.00	9.86	9.73	9.30
**C**	**15–44**				**45–64**				**65–74**				**75–84**			
**Age-specific mortality rates ratio**	**1981**	**1991**	**2001**	**2012**	**1981**	**1991**	**2001**	**2012**	**1981**	**1991**	**2001**	**2012**	**1981**	**1991**	**2001**	**2012**
**Significantly higher than 1**																
Number of municipalities	0	1	5	2	2	4	20	9	4	2	1	12	4	5	5	5
Percentage	0.0%	0.1%	0.6%	0.3%	0.3%	0.5%	2.6%	1.2%	0.5%	0.3%	0.1%	1.6%	0.5%	0.6%	0.6%	0.6%
**Not significantly different from 1**																
Number of municipalities	760	715	763	768	704	458	632	754	711	392	557	745	750	533	576	758
Percentage	98.6%	92.8%	99.0%	99.6%	91.3%	59.4%	82.0%	97.8%	92.2%	50.8%	72.3%	96.6%	97.3%	69.2%	74.8%	98.4%
**Significantly lower than 1**																
Number of municipalities	11	55	3	1	65	309	119	8	56	377	213	14	17	233	190	8
Percentage	1.4%	7.1%	0.4%	0.1%	8.4%	40.1%	15.4%	1.0%	7.3%	48.9%	27.6%	1.8%	2.2%	30.2%	24.6%	1.0%
